# Exploring the Potential Human Kinase-Viral Substrate Network of West Nile Virus

**DOI:** 10.3390/v18080825

**Published:** 2026-07-27

**Authors:** Akash Anil, Ayisha A. Jabbar, Vineetha Shaji, Mukhtar Ahmed, Bristow Ben Joseph, Aromal Monipillil Ajayakumar, Prashant Kumar Modi, Abhithaj Jayanandan, Sowmya Soman, Yashwanth Subbannayya, Rajesh Raju

**Affiliations:** 1 Centre for Integrative Omics Data Science (CIODS), Yenepoya (Deemed to be University), Mangalore 575018, India; akasha.ciods@yenepoya.edu.in (A.A.); ayishaajabbar.ciods@yenepoya.edu.in (A.A.J.); vineethashaji@yenepoya.edu.in (V.S.); bristowben987@gmail.com (B.B.J.); aromalma7645@gmail.com (A.M.A.); abhithaj.j.ciods@yenepoya.edu.in (A.J.); sowmyasoman.ciods@yenepoya.edu.in (S.S.); 2 Department of Zoology, College of Science, King Saud University, Riyadh 11451, Saudi Arabia; mahmed1@ksu.edu.sa; 3 Center for Systems Biology and Molecular Medicine (CSBMM), Yenepoya Research Centre, Yenepoya (Deemed to be University), Mangalore 575018, India; prashantmodi@yenepoya.edu.in; 4 School of Biosciences, Faculty of Health and Medical Sciences, University of Surrey, Guildford GU2 7XH, UK; y.subbannayya@surrey.ac.uk

**Keywords:** West Nile virus, kinase-substrate phosphomotifs, phosphorylation, molecular docking

## Abstract

West Nile virus (WNV) is a mosquito-borne pathogen of escalating epidemiological importance and a growing global health concern, driven by the climate-associated expansion of its *Culex* mosquito vectors. Although WNV is an extensively studied flavivirus, most host–pathogen interaction studies focus on static and structural aspects rather than dynamic and functional ones. Delineating phosphorylation-mediated interactions between WNV proteins and human kinases bridges a critical gap by providing important insight into the molecular mechanisms underlying infection. In this study, we investigated potential phosphorylation-mediated interactions between WNV proteins and human kinases using an integrative computational framework combining motif prediction, phosphoproteomic data analysis and structural docking. Key interactions were predicted between viral proteins and regulatory kinases within the AKT-ERK pathway and the AMPK-mediated autophagy, including major network kinases such as RAF1, IKBKB, and ULK1. In addition, experimentally validated phosphorylation sites in viral proteins were found to be associated with multiple candidate host kinases, including MAP2K7 and MAP2K9, suggesting complex regulatory networks. Integration with phosphoproteomic datasets supported the relevance of multiple predicted kinases, including those associated with antiviral responses and translational regulation. Protein–protein docking demonstrated stable, energetically favorable interactions between selected host kinases and viral proteins, particularly the viral polymerase (NS5), helicase (NS3), and NS1. The findings of this study establish a framework for future research on the development of host-directed antiviral strategies.

## 1. Introduction

West Nile virus (WNV) is a neurotropic flavivirus transmitted by an enzootic cycle comprising birds as natural reservoirs and *Culex* mosquitoes as vectors [1]. In recent decades, WNV has emerged as a significant global concern due to sporadic outbreaks. Its transmission across multiple continents gave rise to distinct regional lineages. Since its first recognized outbreak in Israel in 1951, involving around 100 reported cases, WNV has spread across Africa, Europe, Asia, the Middle East, and the Americas [2]. In 1999, a major outbreak was reported in the United States, with more than 51,000 confirmed cases, and over 2300 deaths, with nearly 50% of infections presenting as neuroinvasive disease involving the central nervous system [3]. In Europe alone, health authorities reported 2083 human cases during the 2018 outbreak. More recently, the 2022 European outbreak caused 1340 locally acquired infections and 104 deaths [4]. Around 20% of infections develop febrile illness, while less than 3% progress to neurological complications such as encephalitis, meningitis, or acute flaccid paralysis [5]. Long-term effects, including cognitive impairment, muscle weakness, fatigue, and motor dysfunction, can persist for years, particularly in elderly and immunocompromised individuals [2]. Hospitalization for long-term neurological care, vector-control programs, and surveillance efforts have imposed a considerable economic burden associated with WNV. Climate change extends mosquito breeding seasons, thereby enhancing the geographic expansion of *Culex* mosquito vectors and increasing the risk of human exposure to West Nile virus [6].

Lineage 1 is the most widespread and is responsible for major outbreaks, including the 1999 U.S. neuroinvasive outbreak, while lineage 2 is mainly found in sub-Saharan Africa and is less virulent. Lineages 3 and 4 are rare and geographically restricted to Europe and Russia. Kunjin virus is an Australian subtype of WNV, which belongs to lineage 1, and lineage 7 (Koutango virus) represents a highly pathogenic strain. Phylogenetic analyses have shown that most Indian WNV isolates belong to a distinct lineage, designated lineage 5, highlighting the long-term circulation and unique evolutionary characteristics of WNV strains in India [7]. Other lineages (6, 8, and 9) are rare and poorly characterized [8,9,10,11,12,13,14]. WNV possesses a single-stranded, positive-sense RNA genome that encodes three structural and seven non-structural proteins. The structural proteins include the capsid protein, which inhibits the serine/threonine protein phosphatase type 2A (PP2A) and helps the virus by degrading claudin proteins and disrupting the epithelial barrier [15]. The pre-membrane/membrane (prM/M) protein protects the virions from fusion with the acidic vesicles of the Golgi complex [16]. The envelope (E) protein facilitates viral entry via clathrin-mediated endocytosis initiated by receptor-mediated attachment. The E protein forms surface spikes in association with the prM protein, and host-mediated prM cleavage promotes virion maturation and antibody resistance. Endosomal acidification induces E protein rearrangement, enabling membrane fusion and release of the viral genome [17]. The WNV non-structural proteins NS1, NS2A, NS2B, NS3, NS4A, NS4B, and NS5 govern viral replication and mediate pathogenesis by interfering with the host immune response. NS1 is essential for viral replication and inhibition of Toll-like receptor 3 (TLR3) signaling, whereas NS2A exhibits immunomodulatory activity [18,19]. NS3 is a conserved protein whose N-terminal domain functions as a trypsin-like serine protease, which becomes catalytically active only when tethered to its essential cofactor, NS2B. While the protease is vital for polyprotein processing, the C-terminal helicase domain of NS3 primarily inhibits alpha/beta interferon signaling. Other enzymatic activities, such as nucleoside triphosphatase and RNA triphosphatase, are also present within its sequence [20]. NS4A is a hydrophobic protein that has been localized in the virus-induced complexes. It carries multiple transmembrane domains and functions closely with NS2A and NS4B in the inhibition of interferon signaling in the flavivirus-infected cells [21]. NS4B plays a major role in the inhibition of interferon signaling by preventing the phosphorylation and accumulation of Janus kinase 1 (JAK1) and tyrosine kinase 2 (Tyk2) [22]. NS5 is the largest protein encoded by the virus with multiple enzymatic activities. The N-terminal of NS5 encodes methyltransferase, and the C-terminal encodes the viral RNA-dependent RNA polymerase for replication of the viral genome [23].

WNV replication is critically dependent on the manipulation of host cellular pathways [24]. Host–virus–interactome studies have revealed that WNV exploits key host antiviral pathways, including the exon-junction complex and nonsense-mediated decay machinery, to protect viral RNA and promote efficient infection [25]. In addition, emerging evidence highlights the important role of lncRNAs in regulating host antiviral responses and virus–host interaction networks during viral infections [26]. Post-translational modifications, such as phosphorylation and glycosylation of viral proteins, have a vital role in the infection cycle of WNV [27,28]. Phosphorylation of the capsid protein reduces RNA binding and oligomerisation while promoting nuclear localisation, thereby acting as an early regulatory mechanism that supports host–virus interactions. As infection progresses, dephosphorylation restores RNA binding and enables efficient virion release. Phosphatidylinositol 3-kinase (PI3K) signaling coordinates the interferon response during WNV infection, revealing distinct mechanistic actions of different PI3K inhibitors on host immunity [29]. WNV also targets the host signaling cascades to promote infection. The Protein Kinase B (AKT) and Extracellular signal-Regulated Kinase (ERK) pathways are activated during early infection in neuronal cells, leading to phosphorylation of downstream effectors, including ribosomal protein S6 (S6), eukaryotic translation initiation factor 4E-binding protein 1 (4E-BP1), and 90 kDa ribosomal S6 kinase (p90RSK). Interestingly, inhibition of ERK or AKT signaling leads to enhanced viral replication, suggesting that the virus transiently activates these pathways to modulate host responses. However, their sustained activity may exert antiviral pressure by inducing cytokines, such as IL-11 [30]. A study by Walter et al. (2025) has revealed the phosphorylation events in the human proteome and WNV proteome during the infection [31]. The study also identified the activation of kinases P21-activated kinase 2 (PAK2) and AMP-activated protein kinase (AMPK) β1, which are associated with antiviral activity [31]. Experimental evidence has shown that a broad spectrum of Protein kinase C (PKC) inhibitors has suppressed WNV replication in vitro, underscoring host kinases as promising yet underexplored antiviral targets in WNV infections.

The development of effective WNV vaccines remains challenging because achieving sufficient viral attenuation while maintaining strong and durable immunogenicity has proven difficult for many flaviviruses [32]. Although several human vaccine candidates for WNV have progressed to preclinical and phase II trials, no approved vaccine or specific antiviral therapy currently exists [33,34]. Certain compounds, such as sofosbuvir, natural isoflavones, and certain antibiotics, have shown *in vitro* anti-WNV activity, but they lacked consistent in vivo efficacy and clinical translation [35]. This therapeutic gap highlights the need to target host perturbations induced by WNV rather than viral components alone. Kinase inhibitors have recently been recognized as potential antivirals with broad and target-specific effects [36,37]. Targeting virus-induced changes in the human phosphoproteome offers an effective approach. [38,39]. Here, we employ computational prediction and phosphomotif analysis to identify host kinases targeting viral proteins. By integrating phosphoproteomic data and host kinase activity profiles during infection, we aim to elucidate dysregulated human kinases associated with WNV infection. This approach enhances our understanding of WNV pathogenesis and supports antiviral development, with kinase-targeting strategies offering dual benefits of limiting viral replication and reducing encephalitis.

## 2. Methodology

### 2.1. Human Kinase-Specific Motif Screening

A systematic kinase–substrate target selection study conducted by Poll B.G. et al. (2024) resulted in a set of kinase–substrate motifs. Poll B.G. et al. (2024) generated visual representations of substrate preferences for 385 kinases using PTM-Logo software (v1.0; ESBL, NIH), developed based on the data generated by Sugiyama et al. (2019), in which they identified 175,574 potential direct substrates and elucidated phosphorylation patterns for 385 recombinant human protein kinases [40,41]. Out of the 384 human kinase–substrate sequence motif logos, 330 statistically significant motifs were chosen for further analysis.

These large-scale analyses have substantially advanced our understanding of kinase–substrate specificities. Johnson et al. (2023) profiled 303 human serine/threonine kinases using synthetic peptide libraries, covering over 84% of the catalytically active serine/threonine kinome, and identified nearly 90,000 phosphorylation sites [42]. In a complementary study, Yaron-Barir et al. (2024) applied combinatorial peptide array screening to map the substrate sequence preferences of all human tyrosine kinases [43]. Their findings demonstrated notable variation in the amino acid patterns surrounding phosphorylation sites and revealed the structural organization of the tyrosine kinome based on motif selectivity [43]. Building on these resources, our analysis applied a 90th percentile threshold to kinase and substrate scores to predict potential kinase–substrate motif associations within WNV proteins. This stringent cutoff was selected to retain high-scoring motif matches and prioritize candidate kinase–substrate interactions for downstream analyses, guided by the scoring framework reported by Johnson et al. (2023) demonstrating enrichment of validated kinase–substrate relationships at high percentile rankings [42].

### 2.2. Mapping Potential Human Kinase Targets Within the WNV Proteome

The entire WNV proteome was retrieved from UniProt (Taxon ID: 11082) [44]. To identify possible host kinase interactions with WNV substrates, established human kinase-specific substrate phosphomotif sequences were cross-referenced against the WNV proteome sequences. Comparative analyses were performed for motif-based prediction using a 7-mer pattern derived from Johnson et al. (2023) and Yaron-Barir et al. (2024) using 80th, 85th, 90th, and 95th percentile cutoffs, and the resulting kinase predictions were compared across thresholds [42,43]. Clustal Omega 2.1 was used to carry out multiple sequence alignment, which enabled the accurate identification of partial sequences while simultaneously assessing the variability and conservation of phosphorylation site motifs [45]. The motif conservation was subsequently evaluated by analyzing amino acids within a −5 to +5 residue window, and the identified kinase–substrate motifs were further investigated for their potential association with kinase activity.

To determine whether the predicted kinases or their associated signaling pathways have been reported in WNV infection, a literature search was conducted. Pathway mapping was performed using the KEGG database (Release 118.0) to contextualize kinase-associated signaling networks [46]. The identified kinases were then compared with those predicted through motif-based analysis. Overlapping kinases were further validated using host phosphoproteomics datasets to determine whether they were experimentally detected during infection, thereby implying their involvement in WNV-associated signaling perturbations.

To predict potential phosphorylation sites and their corresponding kinases within the WNV proteome, two neural network-based tools were also employed: NetPhos, which provides kinase-specific predictions from protein sequences, and GPS/GasPhos, a machine-learning platform for kinase–substrate site prediction [47,48]. Only phosphorylation sites with a prediction score greater than 0.5 were retained for further analysis, to ensure consistency with the scoring framework of the respective tools and to decrease the false positive predictions. The genome polyprotein sequence of WNV strain NY-99 (Taxonomy ID: 1968826) was used as the reference sequence for prediction, as this strain has been widely used in functional studies of WNV proteins [49].

### 2.3. Global Phosphoproteomic Landscape and Phosphorylation Dynamics in Human Cells During WNV Infection

The effect of human kinases on WNV infectivity was investigated using a quantitative differential dataset from the study by Walter et al. (2025) [31]. Differential phosphoproteomics datasets specific to the WNV-infected versus uninfected conditions were assembled using the cutoff criteria (*p*-value < 0.05; fold change: upregulated ≥ 1.3 and downregulated ≤ 0.76), which were previously employed in a study by Shaji et al. (2025) [50]. Further quantitative phosphoproteomic data related to WNV infection were integrated, with phosphorylation profiles assessed based on intensity measurements. All individual phosphosites were then mapped to their corresponding UniProt accessions (downloaded June 2024) via a custom mapping tool to ensure uniform annotation across the data.

Phosphorylation sites in WNV viral proteins associated with infection and pathogenesis were also compiled from studies by Walter et al. (2025) and Julie A. Keating et al. (2013), which reported phosphorylation events within the WNV methyltransferase domain [31,51]. These datasets were processed using the same criteria applied to host phosphoproteomic analysis. Individual phosphosites were mapped to the reference WNV (strain NY-99; Taxon ID: 1968826) using a custom mapping approach based on peptide window sequences derived from phosphorylation data.

### 2.4. Host Kinase Phosphosite Analysis Induced by WNV Infection

To explore the functional significance of the predicted kinases during WNV infection, an expression-based study was performed using phosphoproteomic data. The activation and inhibition profiles of the identified kinases and their WNV-altered phosphosites were then assessed by integrating regulatory site data acquired from the PhosphoSitePlus v6.8.2 database [52].

### 2.5. Structural Assessment of Human Protein Kinases and WNV Proteins

WNV protein structures retrieved from the RCSB Protein Data Bank (RCSB PDB) include WNV NS2B-NS3 protease (PDB ID: 8CO8), WNV envelope glycoprotein (PDB ID: 3I50), RNA-dependent RNA polymerase domain of WNV (PDB ID: 2HFZ), NS5 of WNV (PDB ID: 3LKZ), and NS1 of WNV (PDB ID: 4O6D) [53]. The human kinase structures were also retrieved, including Casein kinase I isoform delta (CSNK1D) (PDB ID: 7P7F), inhibitor of nuclear factor kappa-B kinase subunit beta (IKBKB) (PDB ID: 4KIK), dual specificity mitogen-activated protein kinase kinase 7 (MAP2K7) (PDB ID: 9HZ0), 5′-AMP-activated protein kinase catalytic subunit alpha-1 (PRKAA1) (PDB ID: 4RED), RAF proto-oncogene serine/threonine-protein kinase (RAF1) (PDB ID: 9AY7), serine/threonine-protein kinase Unc-51-like autophagy-activating kinase 1 (ULK1) (PDB ID: 4WNO), and P21-activated kinase 2 (PAK2) (PDB ID: 9D51). The retrieved structures were prepared using OPLS2005. The phosphosites predicted in the unmodelled region of the viral sequence were not included in the analysis.

Protein–protein docking was performed using BioLuminate (version 2024-4) in the Schrodinger suite to investigate the structural interaction between selected human kinases and WNV proteins [50]. This analysis aimed to explore potential interaction interfaces and evaluate the spatial proximity between kinase active sites and viral protein regions, providing structural insights into kinase–viral protein associations. The potential human kinases were selected as receptors, and the WNV proteins served as ligands. The catalytic domain of the kinases and putative phosphosites of the corresponding viral proteins were defined in the docking. Thirty protein complex poses were generated and ranked based on the PIPER cluster size and PIPER pose energy, which evaluates receptor–ligand interactions and is efficiently computed using Fast Fourier Transforms [54]. The top-scoring complex was selected for further analysis. The protein interaction analysis module in Biologics (Schrödinger 2025-3) was utilized to determine kinase–protein interactions and to examine the interaction status of the sites predicted in our analysis.

### 2.6. Molecular Dynamics Simulation

**Figure 1:**
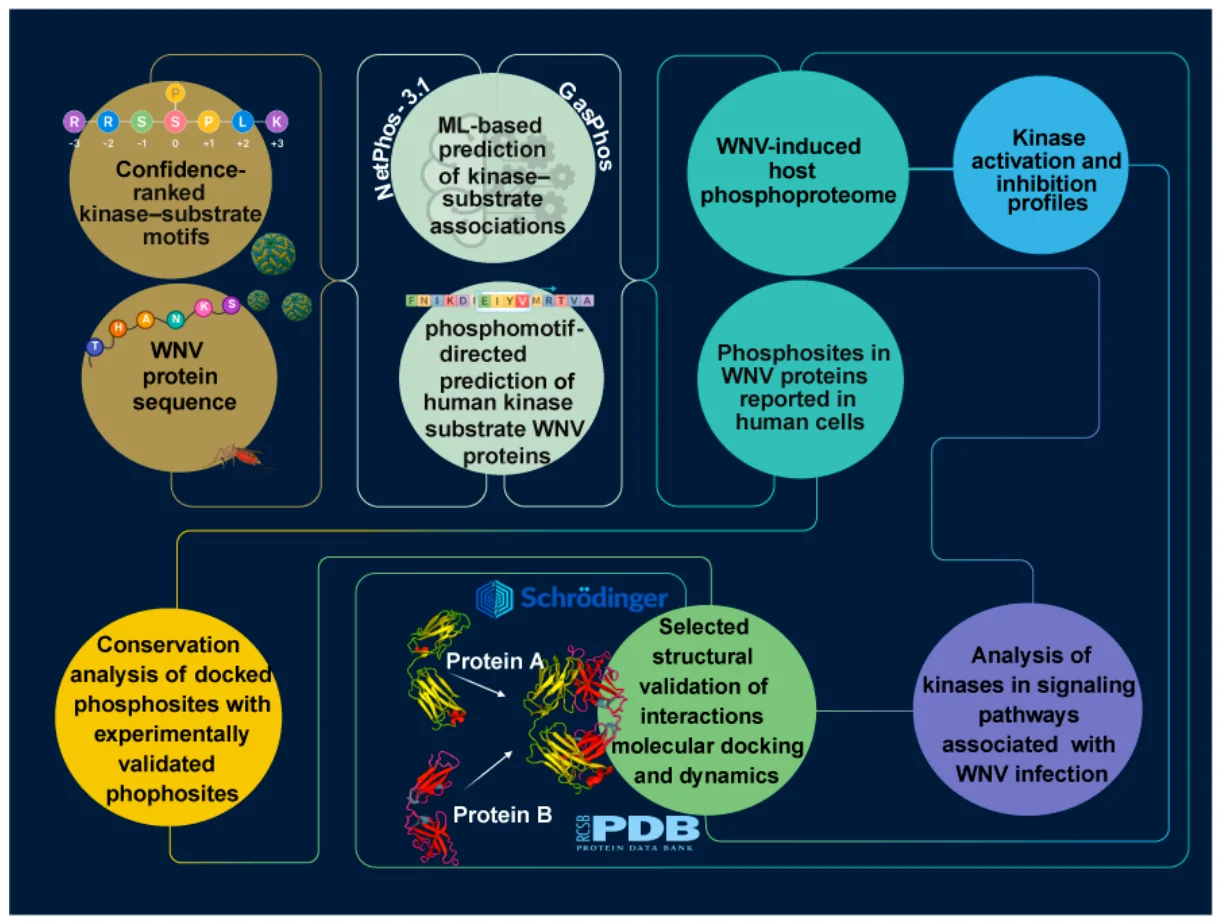
Workflow of the study.

Molecular dynamics (MD) simulations were conducted using the Desmond module of the Schrödinger suite (Schrödinger Maestro 2024-3). The selected protein–protein complexes identified from the protein–protein docking analysis were subjected to MD simulations. Each system was solvated in an orthorhombic simulation box containing TIP3P water molecules, and appropriate numbers of Na^+^ and Cl^−^ ions were added using the System Builder tool to neutralize the system. Energy minimization was carried out employing the OPLS4 force field. Subsequently, 100 ns production simulations were performed under NPT ensemble conditions at 300 K and 1.013 bar. The temperature and pressure were regulated using the Nosé–Hoover chain thermostat and the Martyna–Tobias–Klein barostat, respectively. Protein–protein interaction analysis was performed to evaluate the stability and dynamic behavior of the complexes. After completing the simulations, trajectory analyses were performed, including the calculation of root mean square deviation (RMSD) and root mean square fluctuation (RMSF). The entire workflow of the study is illustrated in Figure 1.

## 3. Results

### 3.1. Human Kinase-Target Motifs in WNV Proteome

We retrieved 5238 protein sequences from UniProt for different WNV strains and isolates. The motif-based prediction using a 7-mer pattern derived from the Johnson et al. (2023) [42] and Yaron-Barir et al. (2024) [43] identified 300 potential human kinases (16 dual-specificity, 8 tyrosine, and 276 serine/threonine) that can phosphorylate WNV proteins. The 9-mer pattern motif search, following the same study, identified 93 potential kinases (87 serine/threonine and 6 dual-specificity). Briefly, 41 potential kinases were predicted utilizing Poll B.G. et al. (2024) motifs, including 23 serine/threonine, 13 tyrosine kinases and 5 dual specificity. The predicted kinases and corresponding phosphosites utilizing the motif-based search are provided in Supplementary Tables S1–S6. Comparative analysis demonstrated that several key kinases including RAF1, CSNK1D, PAK2, ULK1, and MAP2K2, were consistently identified across the 80th, 85th, 90th, and 95th percentile thresholds (Supplementary Table S11).

Computational tools, NetPhos predicted 12 kinase families for 295 potential phosphorylation sites, and GasPhos predicted 21 kinases for 430 potential phosphorylation sites. Both tools predicted the Ataxia Telangiectasia Mutated (ATM), epidermal growth factor receptor (EGFR), protein kinase A (PKA), and PKC kinase families at multiple sites. The predicted kinases and corresponding phosphosites from computational tools are provided in Supplementary Tables S7 and S8.

### 3.2. Host Phosphorylation Landscape and Signaling Alterations Induced by WNV Infection

**Figure 2:**
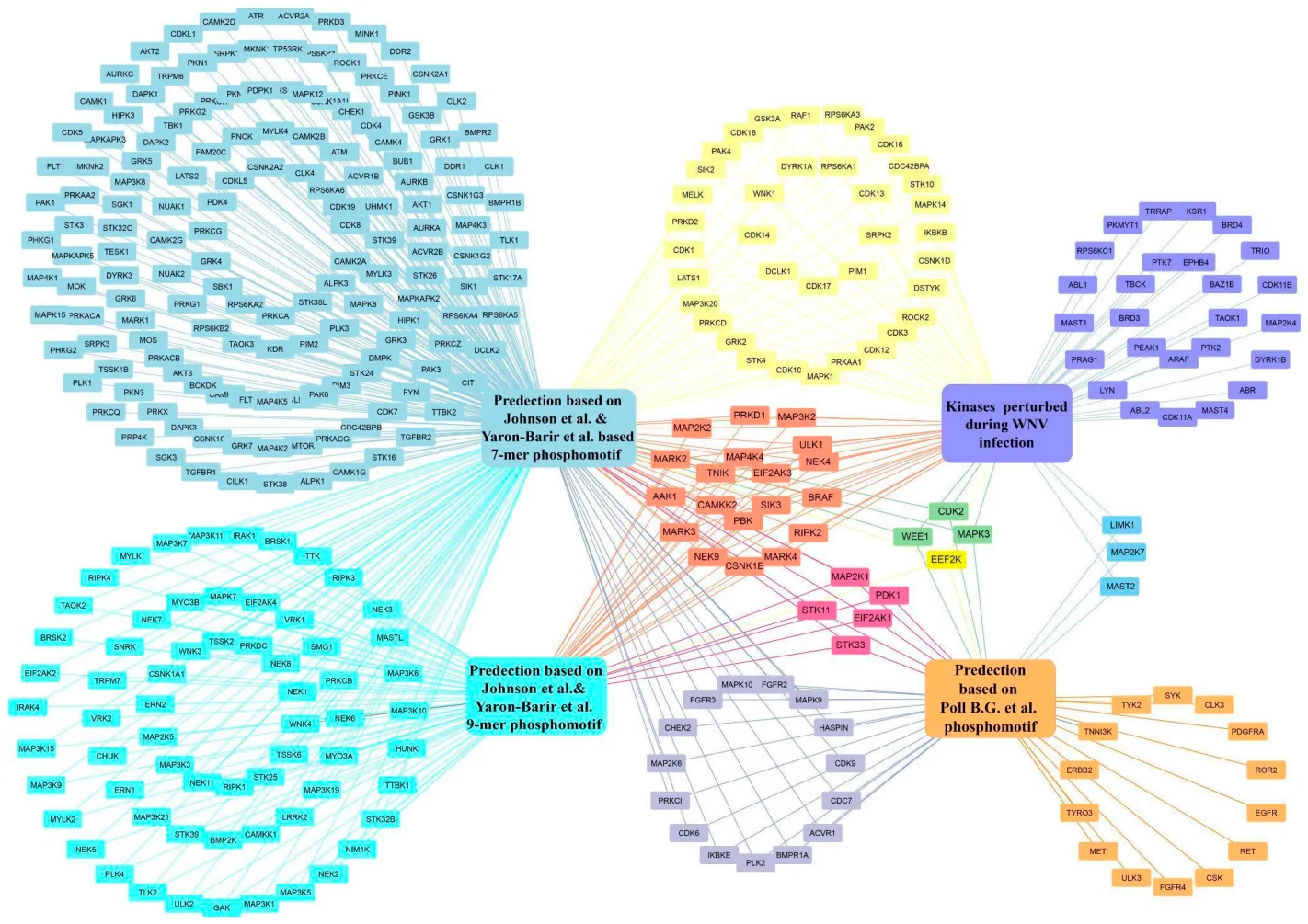
Network representation of host kinases predicted using motif-based approaches and kinases perturbed during WNV infection. Distinct color-coded clusters represent kinases common across prediction methods and perturbed kinases [40,42,43].

Analysis of the differential phosphoproteomic datasets from Walter et al. (2025) [31] revealed significant alterations in host phosphorylation dynamics during WNV infection. Among the 41 kinases predicted based on the kinase–substrate motif study by Poll B.G. et al. (2024), seven kinases (cyclin-dependent kinase 2 (CDK2), eukaryotic elongation factor 2 kinase (eEF2K), LIM domain kinase 1 (LIMK1), mitogen-activated protein kinase 3 (MAPK3), MAP2K7, microtubule-associated serine/threonine kinase 2 (MAST2), and WEE1) overlapped with WNV-infected host phosphoproteomic datasets, with CDK2_Y15 and MAPK3_Y204 previously reported to have regulatory roles [55,56]. At a 7-amino acid confidence threshold, 300 kinases were predicted, including adaptor-associated kinase 1 (AAK1), ULK1, and IKBKB, along with members of major kinase families such as CDKs, MAPKs, AKT/AGC kinases, CAMKs, casein kinases, and tyrosine kinases, of which 39 have documented modulation during WNV infection. Among these, calcium/calmodulin-dependent protein kinase kinase 2 (CAMKK2), cyclin-dependent kinase 1 (CDK1), cyclin-dependent kinase 2 (CDK2), cyclin-dependent kinase 3 (CDK3), G protein-coupled receptor kinase 2 (GRK2), PAK2, and serine-arginine protein kinase 2 (SRPK2) are established regulators of host signaling. Notably, 24 kinases were consistently predicted across both the Poll B.G. et al. (2024) phosphomotif-based analysis and the 7-mer motif-based prediction approach based on the kinase specificity datasets of Johnson et al. (2023) [42] and Yaron-Barir et al. (2024) [43] (Figure 2).

### 3.3. Potential Kinases Associated with Experimentally Validated Sites of WNV Proteins

**Figure 3:**
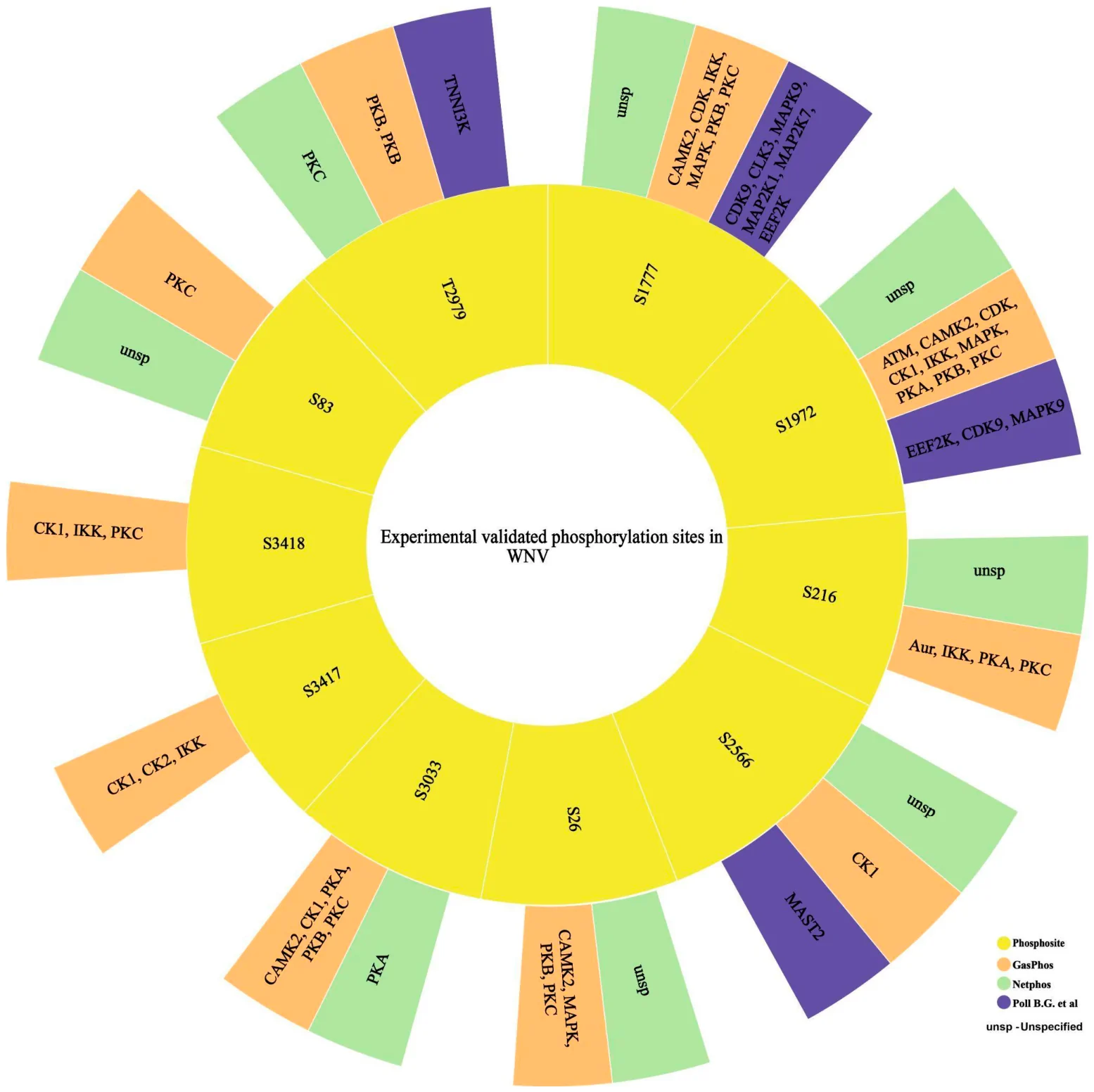
Human kinases predicted corresponding to experimentally validated sites in WNV proteins utilizing Poll B.G. et al. (2024) [40] based motif pattern-prediction, GasPhos [47] and Netphos [48].

Motif pattern analysis identified multiple kinases corresponding to experimentally validated phosphorylation sites in WNV proteins. The kinase–substrate motifs described by Poll B.G. et al. (2024) identified cyclin-dependent kinase 9 (CDK9), CDC-like kinase 3 (CLK3), mitogen-activated protein kinase 9 (MAPK9), mitogen-activated protein kinase kinase 1 (MAP2K1), MAP2K7, and eEF2K for the experimentally validated phosphosite (S1777) in NS3. Similarly, for the site S1972, the predicted kinases included eEF2K, CDK9, and MAPK9. The site S2566 was predicted to be phosphorylated by MAST2, and T2979 phosphorylated by TNNI3K (Figure 3).

Previous studies have reported activation of the MAPK/ERK signaling pathway in response to WNV infection, highlighting its role in host cellular responses and viral pathogenesis. Our motif pattern analysis predicted multiple kinases associated with the ERK signaling pathway. Notably, mitogen-activated protein kinase 1 (MAPK1), mitogen-activated protein kinase kinase 2 (MAP2K2), MAPK3, and RAF1, which are key components of the ERK signaling pathway, were predicted from the +3/−3 motif pattern described by Johnson et al. (2023) [42] and Yaron-Barir et al. (2024) [43]. These kinases were also detected in host phosphoproteomic datasets from WNV-infected samples, supporting their potential involvement in the infection process.

### 3.4. Docking of Predicted Phosphorylation Sites with Potential Kinases

Protein–protein docking analysis of selected human kinases and WNV viral proteins revealed protein complexes with favorable docking parameters. These docking poses were selected for subsequent structural analysis based on their favorable PIPER pose energies and cluster size. The RAF1-NS5 (9AY7_2HFZ) complex exhibited a PIPER pose energy of −1551.240 kcal/mol with a cluster size of 41, suggesting a stable interaction interface between the kinase and the viral RNA-dependent RNA polymerase. Similarly, the IKBKB-NS5 (8T74_2HFZ) complex displayed a PIPER pose energy of −1624.276 kcal/mol with a cluster size of 38, indicating favorable binding between the kinase catalytic region and the NS5 protein. The ULK1-NS5 (4WNO_3LKZ) complex showed a PIPER pose energy of −1286.835 kcal/mol with a cluster size of 99, suggesting consistent docking conformations across multiple poses. The MAP2K7-NS3 (9HZ0_2QEQ) complex exhibited a PIPER pose energy of −1405.327 kcal/mol with a cluster size of 82, suggesting a favorable interaction between the kinase and the viral helicase protein. Among the complexes analyzed, the PRKAA1-NS5 (4RED_2HFZ) interaction exhibited one of the most favorable PIPER pose energies, −1812.936 kcal/mol, with a cluster size of 54, suggesting a highly stable and well-populated docking interface. Similarly, the PAK2-NS1 (9D51_4O6D) complex demonstrated a PIPER pose energy of −1889.949 kcal/mol with a cluster size of 60, indicating a strong and consistent interaction cluster. CSNK1D-NS5 (7P7F_2HFZ) complex demonstrated a PIPER pose energy of −1407.259 kcal/mol and cluster size of 22 (Supplementary Table S9). The lower PIPER pose energies indicate more favorable docking solutions within the scoring framework; these values primarily serve to prioritize candidate interaction interfaces for further analysis.

### 3.5. Interaction Analysis Between the Phosphorylation Sites in WNV and Human Kinases

**Figure 4:**
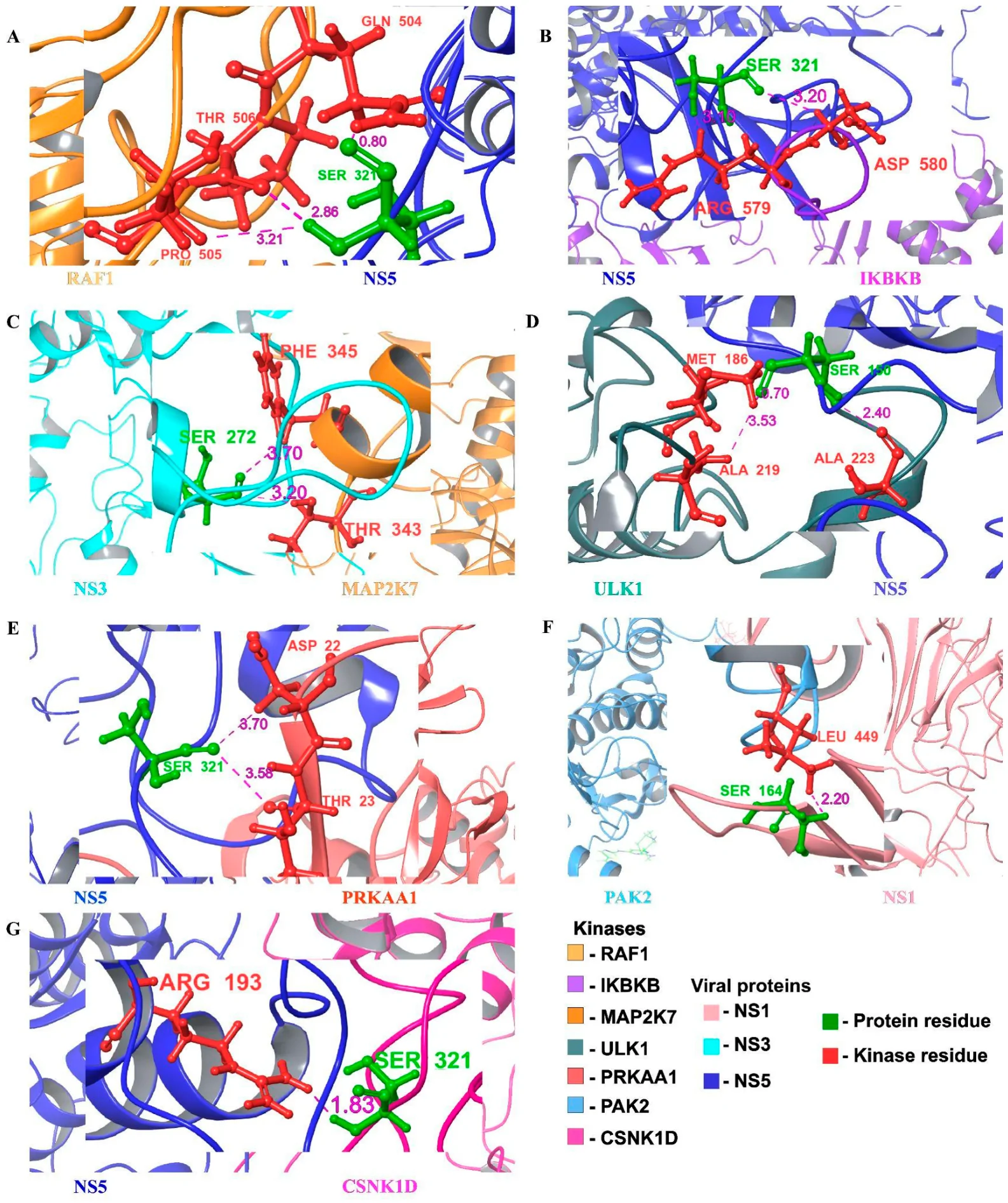
Interaction between predicted phosphosites and kinase domain residues of predicted potential human kinases. (**A**) Interaction between S321 in NS5 and kinase domain residues of RAF1. (**B**) Interaction between S321 in NS5 and kinase domain residues of IKBKB. (**C**) Interaction between S272 in NS3 and kinase domain residues of MAP2K7. (**D**) Interaction between S150 in NS5 and kinase domain residues of ULK1. (**E**) Interaction between S321 in NS5 and kinase domain residues of PRKAA1. (**F**) Interaction between S164 in NS1 and kinase domain residues of PAK2. (**G**) Interaction between S321 in NS5 and kinase domain residue of CSNK1D.

In the RAF1-NS5 complex (9AY7_2HFZ), the predicted phosphorylation site S321 of NS5 was positioned in close proximity to the kinase domain residues T506, Q504, and P505 (Figure 4A). Among these, Q504 formed a hydrogen bond with S321, accompanied by high surface complementarity values (95% for RAF1 and 63% for NS5), indicating a stable interaction interface. In the IKBKB-NS5 complex, kinase domain residues R579 and D580 were located in proximity to the predicted phosphorylation site S321 of NS5 (Figure 4B). The short intermolecular distances and moderate surface complementarity suggest that the S321 region of NS5 remains structurally accessible for potential kinase-mediated phosphorylation. Buried solvent-accessible surface area (SASA) analysis further supports the stability of the interaction between the interacting surfaces. Given that RAF1 and IKBKB function as upstream regulators of the MEK-ERK signaling cascade, this interaction may provide a structural basis for potential modulation of ERK pathway signaling during WNV infection [57,58].

Within the MAP2K7_NS3 complex kinase domain, residues F345 and T343 were located in proximity (3.2–3.7 Å) to the predicted phosphorylation site S272 (Figure 4C). These residues exhibit moderate surface complementarity (~0.46) and high residue burial (>90% buried SASA), indicating their potential contribution to the interaction interface. Within the ULK1-NS5 complex, S150 of NS5 formed a hydrogen bond with A223 of ULK1, with buried SASA values of 76% for ULK1 and 73% for NS5, indicating a stable interaction interface. In addition, S150 displayed close proximity and van der Waals contacts with A219 and M186, suggesting that this region of NS5 may represent a structurally favorable interaction site (Figure 4D).

Similarly, in the PRKAA1_NS5 complex, the predicted phosphorylation site, S321, was located near the kinase domain residues N22 and T23. These contacts occur at intermolecular distances of 3.6–3.7 Å with moderate surface complementarity (0.32–0.50), accompanied by substantial burial of interacting residues, particularly NS5 S321 (~91% buried SASA) (Figure 4E). The interaction appears to be mediated primarily by van der Waals contacts and surface packing. In the PAK2_NS1 complex, the predicted phosphorylation site S164 was located in proximity to the kinase domain residue L449, with a short intermolecular distance of 2.2 Å and high surface complementarity (0.82), suggesting a favorable geometric interaction interface (Figure 4F). Notably, in the CSNK1D_NS5 complex, S321 formed a hydrogen bond with R193 (1.8 Å), with moderate surface complementarity (~0.6) and high residue burial (>84% SASA), supporting a defined interaction (Figure 4G). The relevance of the kinases selected for docking analysis is given in Table 1. All the interaction between human kinase and WNV protein is given in Supplementary Table S10.

### 3.6. Molecular Dynamics Simulation

#### 3.6.1. RMSD

Molecular dynamics simulation of the RAF1–NS5 complex demonstrated overall structural stability throughout the simulation period. The protein backbone RMSD stabilized after the initial equilibration phase and remained within an acceptable range (~2.5–3.8 Å), indicating maintenance of the docked complex architecture. These findings provide additional dynamic support for the proposed RAF1–NS5 interaction interface identified through docking analyses. The ULK1-NS5 complex underwent an initial equilibration phase followed by stabilization of backbone RMSD values around 2–3 Å throughout the simulation, indicating maintenance of the overall docked conformation under dynamic conditions. These findings provide additional structural support for the proposed interaction interface.

#### 3.6.2. Protein–Protein Interactions

**Figure 5:**
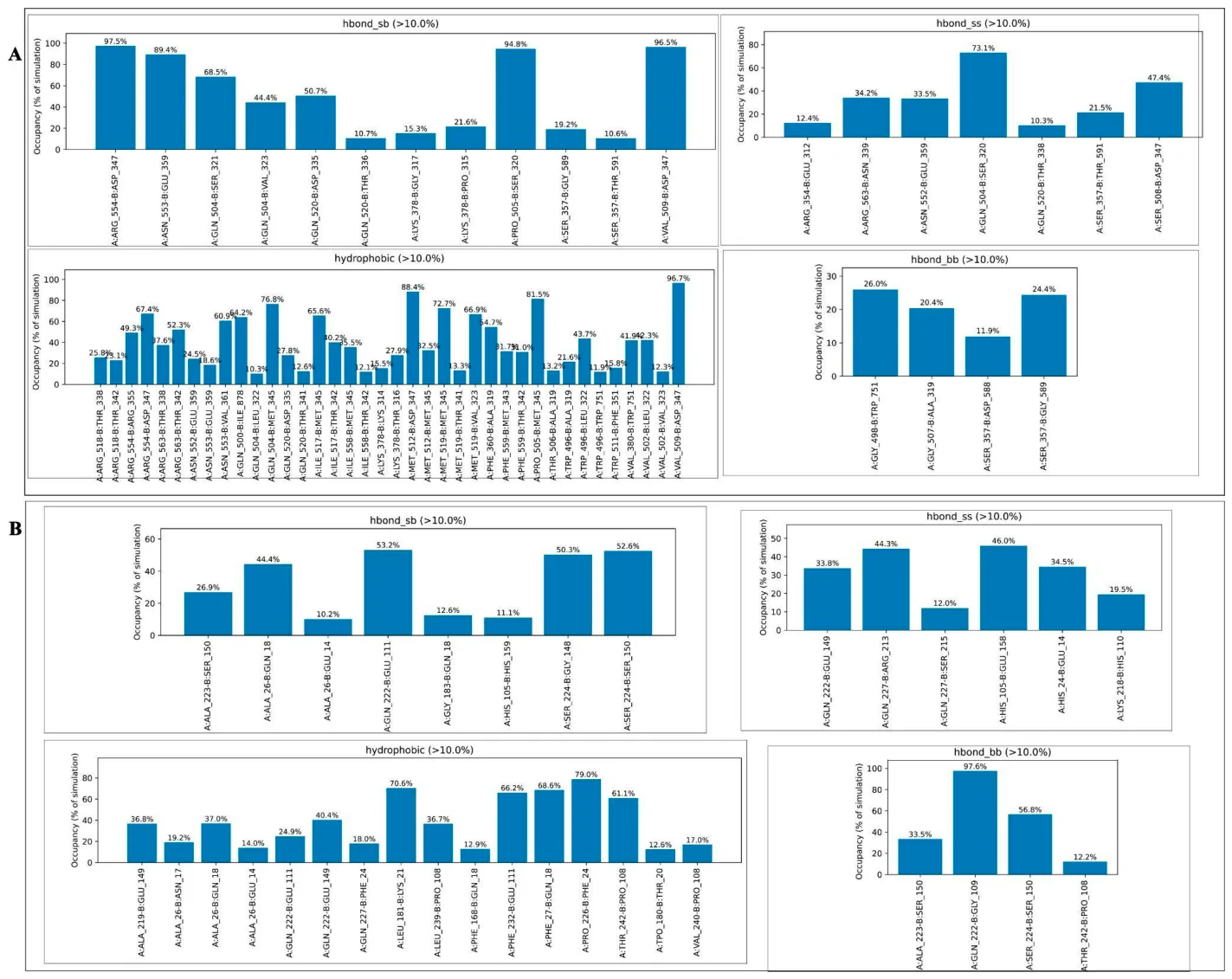
Protein interactions during MD simulation. (**A**) Protein interactions of interacting residues in RAF1–NS5 complex. (**B**) Protein interactions of interacting residues in ULK1-NS5 complex.

Interaction occupancies were calculated throughout the simulation trajectory, and only contacts persisting for more than 10% of the simulation time were considered. Molecular dynamics simulations further supported the stability of the RAF1-NS5 complex. Interaction occupancy analysis revealed several persistent intermolecular contacts throughout the simulation. Notably, the GLN504-SER321 hydrogen bond identified in the docking model was maintained for a substantial portion of the trajectory, supporting the proposed interaction interface. In addition, multiple hydrophobic contacts and hydrogen-bonding interactions remained stable during the simulation, while salt bridges and water-mediated interactions further contributed to interface stabilization (Figure 5A). Similarly, the interaction occupancy analysis of the ULK1-NS5 complex revealed persistent intermolecular contacts throughout the simulation. Several hydrogen bonds, hydrophobic interactions, and salt-bridge interactions were maintained for a substantial portion of the trajectory, indicating an interaction network at the protein–protein interface. Water-mediated interactions further contributed to complex stabilization (Figure 5B). Overall, the molecular dynamics simulations demonstrated sustained intermolecular contacts and stable interaction networks in both complexes, providing dynamic support for the proposed kinase-NS5 interaction interfaces while acknowledging that experimental validation is required to confirm their relevance.

#### 3.6.3. Root Mean Square Fluctuation (RMSF)

**Figure 6:**
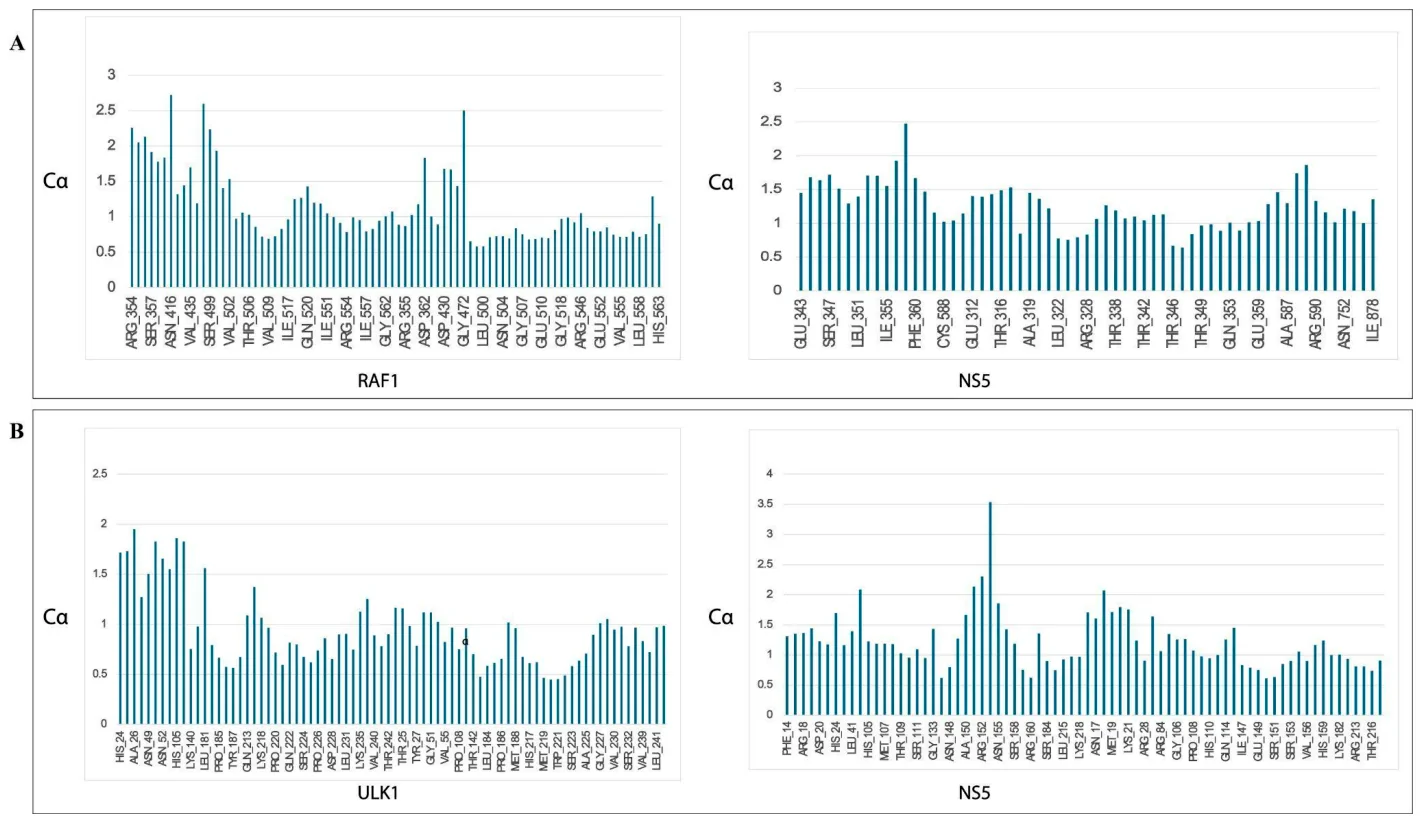
RMSF values of interacting residues in human kinase viral protein complex. (**A**) RMSF values of interacting residues in RAF1–NS5 complex. (**B**) RMSF values of interacting residues in the ULK1-NS5 complex.

Root mean square fluctuation (RMSF) analysis of the interacting residues indicated overall structural stability of the RAF1-NS5 complex throughout the simulation. The interacting residues of RAF1 exhibited low average fluctuations (mean RMSF = 1.14 ± 0.51 Å), while the corresponding NS5 interacting residues showed similarly limited mobility (mean RMSF = 1.26 ± 0.35 Å) (Figure 6A). Most interface residues in both proteins displayed RMSF values below 2.0 Å, suggesting a stable protein–protein interaction with only minor local flexibility. Similarly, RMSF analysis of the ULK1–NS5 complex demonstrated persistent behavior of the protein–protein interface during the simulation. The interacting residues of ULK1 exhibited low average fluctuations (mean RMSF = 0.94 ± 0.36 Å), whereas the interacting residues of NS5 showed slightly higher but still moderate fluctuations (mean RMSF = 1.22 ± 0.47 Å) (Figure 6B). The majority of interface residues in both proteins remained below 2.0 Å, indicating restricted conformational mobility and maintenance of a stable interaction interface throughout the simulation.

### 3.7. Conservation Analysis

**Figure 7:**
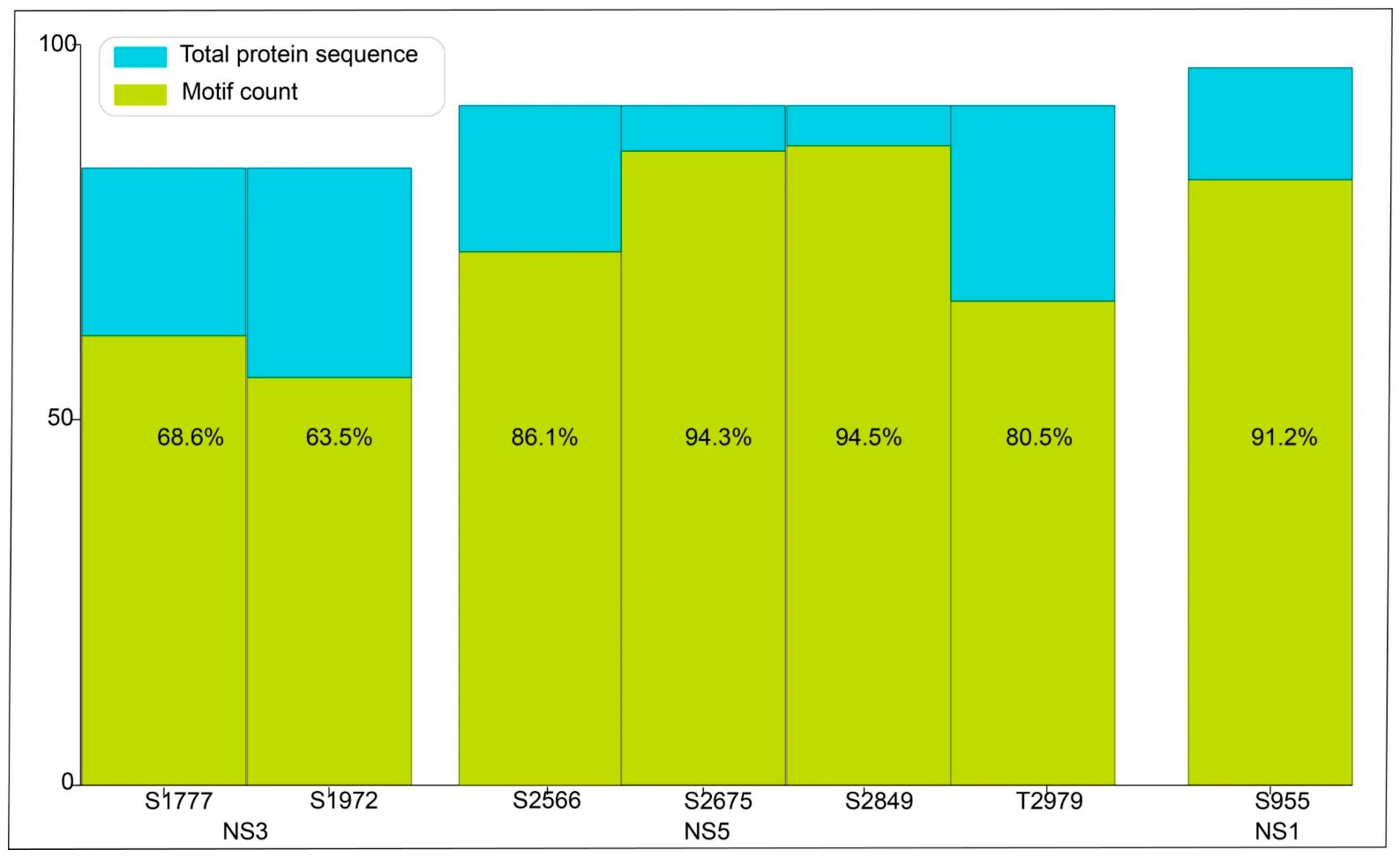
Conservation analysis of experimentally validated phosphorylation sites and structurally supported interaction sites identified through docking.

Conservation analysis of experimentally validated phosphorylation sites reported by Poll B.G. et al. (2024) revealed that S1777 and S1972 exhibit moderate conservation (68% and 63%, respectively) across diverse WNV strains and isolates. Relatively higher conservation was observed in S2566 (73%), while T2979 showed marked conservation (~80%), indicating evolutionary retention. Furthermore, the phosphorylation sites implicated in kinase–substrate interactions in our structural analysis also showed substantial conservation. In particular, S2849 and S2675 within NS5 were highly conserved (~94%), whereas S955 in NS1 was 91% conserved across the analyzed sequences. Collectively, the conservation observed for these predicted phosphorylation sites, especially those supported by structural interaction data, suggests their potential relevance in the WNV life cycle and evolutionary retention. Conserved sites may also reflect structural or evolutionary constraints within viral proteins. Therefore, conservation can be considered supportive evidence for prioritizing candidate phosphorylation sites for further investigation. Experimental studies will be required to further clarify the biological significance of these predicted phosphorylation events (Figure 7).

## 4. Discussion

The neglected tropical pathogen WNV has evolved into a global public health concern, driven by climate change and ecological shifts, and is now one of the leading causes of viral encephalitis. The absence of approved human vaccines or specific antiviral therapies has made it critical to delineate how WNV interacts with host cellular machinery for identifying new therapeutic targets [59]. Thus, elucidating the phosphorylation events on viral proteins can help uncover host pathways exploited during infection and support the discovery of novel host-targeted antiviral strategies [60,61]. Considering the known interference of host phosphosignaling pathways, our study identified multiple host kinases that may participate in these virus–host interactions. Previous studies have shown that activation of the AKT and ERK signaling pathways occurs during WNV infection in neuronal cells. The pharmacological inhibition of these pathways significantly alters viral replication [30]. In line with this, our study predicted multiple kinases associated with the AKT and ERK pathways, including RAF1, IKBKB, MAPK1, MAPK3, and MAP2K2, as potential upstream regulators of WNV proteins. The lack of modeled viral protein structures limited our analysis, restricting docking studies to RAF1 and IKBKB, which showcased interaction with the WNV non-structural protein NS5. This supports the possibility of signaling hijack by WNV to promote viral replication and regulate infection-associated cellular responses. RAF1 has been extensively associated with cancer progression, where its phosphorylation-dependent regulation influences diverse cellular processes, making it an important therapeutic target in cancer and a relevant target against other diseases [62,63].

Experimental evidence has demonstrated that the WNV capsid protein can suppress autophagy and highlighted the importance of the AMPK pathway [64]. Consistent with this observation, phosphoproteomic studies have also reported activation of host kinases, such as AMPK and PAK2 during WNV infection, which was shown to influence antiviral responses and viral genome translation [31]. In agreement with these experimental findings, our computational analysis predicted potential interactions between PRKAA1 (AMPK), PAK2, and WNV proteins. Subsequent docking analyses revealed interactions between PRKAA1 and NS5 and PAK2 and NS1. The phosphorylation site PAK2_S141, perturbed upon WNV infection, was associated with kinase activity. ULK1, which is upstream of AMPK, was also predicted by our analysis, and the subsequent docking showed a reliable interaction between ULK1 and NS5. ULK1 serves as a critical regulator of autophagy initiation by coordinating upstream nutrient-sensing pathways. Together, these observations suggest that suppression of host cellular responses is important for WNV pathogenesis, and autophagy-related kinase pathways may play a significant role in developing novel therapeutic agents. These findings collectively suggest that WNV may strategically target autophagy-associated kinome rewiring to modulate host antiviral defenses and establish a cellular environment favorable for viral survival and replication.

Experimental studies have also demonstrated direct interactions between host kinases and WNV proteins [65]. For instance, the WNV NS5 methyltransferase domain has been shown to interact with protein kinase G (PKG) and undergo phosphorylation during infection, suggesting that host kinases may modulate viral replication through direct protein–protein interactions. Similarly, casein kinase 1 (CK1) has been reported to phosphorylate the flaviviral methyltransferase domain, influencing viral replication processes [51,66]. Notably, CSNK1D and Casein kinase 1 epsilon (CSNK1E) were predicted by our motif-based analysis, and CSNK1D exhibited a favorable interaction with NS5 at S321, characterized by a hydrogen bond with the kinase domain. In addition, the phosphorylation site CSNK1D T347, reported to be induced during WNV infection, has been linked to kinase activation, suggesting that CSNK1D may be functionally engaged during infection [67]. To further evaluate the stability of the predicted kinase–viral protein interactions, molecular dynamics simulations were performed for the RAF1-NS5 and ULK1-NS5 complexes. Both complexes maintained stable interaction interfaces throughout the simulation trajectories, with persistent intermolecular contacts and limited fluctuations at the interacting residues. These observations provide additional support for the proposed human kinase-viral protein associations. AAK1, identified in our motif-based prediction, has been reported as a potential antiviral target, with inhibitors demonstrating activity against multiple viruses, including WNV [68]. AAK1 is known to regulate clathrin-mediated endocytosis, a process critical for viral entry, suggesting that targeting this kinase may disrupt the early stages of infection [69]. Multiple kinases were predicted to target the NS5 residue S321. This observation may reflect overlapping substrate recognition preferences among serine/threonine kinases rather than strict kinase specificity for a single phosphosite. It indicates the potential alternative candidate kinase–substrate relationships. The predicted kinase–viral protein interactions may have broader relevance across flaviviruses. NS5 is a highly conserved multifunctional protein among flaviviruses, including Dengue virus (DENV), Zika virus (ZIKV), Japanese encephalitis virus (JEV), and Yellow fever virus (YFV), and conserved protein interaction interfaces have been reported within flaviviral NS5 proteins [70,71]. In addition the inhibition of RAF-associated signaling has been shown to impair ZIKV replication [72]. While the conservation of the specific kinase–substrate interactions predicted here remains to be experimentally validated, these observations suggest that related host kinase-dependent regulatory mechanisms may operate across multiple flaviviruses. In addition, natural compounds such as lycorine, which exhibit antiviral activity against WNV, have been reported to act as multi-kinase inhibitors targeting pathways including EGFR and MEK signaling [73,74,75]. Together, it suggests WNV may perturb host kinase networks as a strategic mechanism to regulate replication and host response modulation, thereby positioning host kinases as key targets for the development of effective host-directed antiviral interventions. While docking analysis identified structurally plausible interaction interfaces between the predicted kinases and WNV proteins. Future validation efforts of the predicted kinase–substrate associations could employ several methodologies, such as co-immunoprecipitation to assess protein–protein interactions, kinase activity assays to confirm phosphorylation events, and phosphosite-directed mutagenesis to elucidate functional consequences. Additionally, phosphoproteomic investigations may be utilized to confirm specific phosphorylation events occurring in the context of WNV infection.

## 5. Conclusions

The present study provides a robust computational investigation of host–virus interactions in WNV infection by integrating motif-based phosphorylation prediction, phosphoproteomic data evidence, and structural interaction analyses. The findings of the study highlight the involvement of host phosphorylation networks in regulating viral proteins and suggest that multiple cellular signaling pathways may participate in modulating viral replication and host responses during infection. Structural interaction analyses further supported the feasibility of these predicted regulatory interfaces, indicating that viral proteins may interact with host signaling components to influence key stages of the viral life cycle. Collectively, these observations underscore the importance of kinase-mediated phosphoregulation of host and viral proteins in WNV pathogenesis and provide insights into potential host-directed antiviral strategies. Future experimental validation of the predicted interactions and phosphorylation is critical to establish their functional attributes and roles in WNV pathogenesis. Such integrative insights facilitate future comprehensive studies and accelerate the identification of novel intervention strategies targeting host signaling pathways in flaviviral infections.

## Figures and Tables

**Table 1:** Key kinases predicted and their relevance.

Predicted Kinase	WNV Protein	Predicted Phosphosite	Phosphoproteomic Evidence	Pathway/Biological Relevance	Docking Support
RAF1	NS5	S2849	RAF1 overlap with WNV phosphoproteomic dataset	MAPK/ERK signaling	Hydrogen bond with kinase domain residue; high surface complementarity; MD support
PRKAA1	NS5	S2849	PRKAA1 overlaps with the WNV phosphoproteomic dataset.	AMPK/autophagy pathway	Close kinase-phosphosite proximity
IKBKB	NS5	S2849	IKBKB overlap with WNV phosphoproteomic dataset	MAPK/ERK signaling	Catalytic residues positioned near phosphosite
CSNK1D	NS5	S2849	CSNK1D overlap with WNV phosphoproteomic dataset	CK1 family kinase associated with flaviviral replication processes.	Hydrogen bond with kinase domain residue; high residue burial
PAK2	NS1	S955	PAK2 overlap with WNV phosphoproteomic dataset	Antiviral response and viral translation regulation	Close contact with kinase domain; high surface complementarity
MAP2K7	NS3	S272	MAP2K7 overlap with WNV phosphoproteomic dataset	JNK/MAPK signaling pathway	Close phosphosite proximity; favorable interaction interface
ULK1	NS5	S150	ULK1 overlap with WNV phosphoproteomic dataset	Autophagy initiation pathway	Hydrogen bond interaction; MD support

## Data Availability

The study data are available in the article and Supplementary Materials. For further inquiries, contact the corresponding author.

## Supplementary Materials

The following supporting information can be downloaded at https://www.mdpi.com/article/10.3390/v18080825/s1: Supplementary Table S1: Prediction results based on Poll B.G. et al. based on 11 aminoacid centric motif. Supplementary Table S2: Prediction results based on Johnson et al. and Yaron-Barir et al. based on 7 aminoacid centric motif (90th percentile threshold). Supplementary Table S3: Prediction results based on Johnson et al. and Yaron-Barir et al. based on 9 aminoacide centric motif. Supplementary Table S4: Prediction results based on Johnson et al. and Yaron-Barir et al. based on 7 aminoacid centric motif (95th percentile threshold). Supplementary Table S5: Prediction results based on Johnson et al. and Yaron-Barir et al. based on 7 aminoacid centric motif (85th percentile threshold). Supplementary Table S6: Prediction results based on Johnson et al. and Yaron-Barir et al. based on 7 aminoacid centric motif (80th percentile threshold). Supplementary Table S7: Kinases predicted for WNV [strain NY-99 (Taxonomy ID: 1968826)] utilizing Netphos. Supplementary Table S8: Kinases predicted for WNV [strain NY-99 (Taxonomy ID: 1968826)] utilizing Gasphos. Supplementary Table S9: Docking analysis of predicted host kinases with selected WNV proteins. Supplementary Table S10: Interaction analysis between human kinase and WNV protein. Supplementary Table S11: Comparative analysis of kinases identified based on Johnson et al. and Yaron-Barir et al. based on 7 aminoacid centric motif across the 80th, 85th, 90th, and 95th percentile thresholds.

## Abbreviations

WNV	West Nile virus
PP2A	Protein phosphatase type 2A
prM/M	Pre-membrane/membrane
TLR3	Toll-like receptor 3
JAK1	Janus kinase 1
Tyk2	Tyrosine kinase 2
PI3K	Phosphatidylinositol 3-kinase
4E-BP1	4E-binding protein 1
p90RSK	90 kDa ribosomal S6 kinase
ERK	Extracellular signal-regulated kinase
AKT	Protein kinase B
PAK2	P21-activated kinase 2
AMPK	AMP-activated protein kinase
CSKN1D	Casein kinase 1 isoform delta
IKBKB	Inhibitor of nuclear factor kappa-B kinase subunit beta
MAP2K7	Mitogen-activated protein kinase kinase 7
PRKAA1	Protein kinase catalytic subunit alpha-1
RAF1	RAF proto-oncogene serine/threonine-protein kinase
ULK1	Unc-51-like autophagy-activating kinase 1
ATM	Ataxia Telangiectasia Mutated
EGFR	Epidermal growth factor receptor
PKA	Protein kinase A
CDK2	Cyclin-dependent kinase 2
eEF2K	Eukaryotic elongation factor 2 kinase
LIMK1	LIM domain kinase 1
MAPK3	Mitogen-activated protein kinase 3
MAST2	Microtubule-associated serine/threonine kinase 2
AAK1	Adaptor-associated kinase 1
CAMKK2	Calcium/Calmodulin-dependent protein kinase kinase 2
CDK1	Cyclin-dependent kinase 1
CDK3	Cyclin-dependent kinase 3
GRK2	G protein-coupled receptor kinase 2
SRPK2	Serine-arginine protein kinase 2
CDK9	Cyclin-dependent kinase 9
MAPK9	Mitogen-activated protein kinase 9
MAP2K1	Mitogen-activated protein kinase kinase 1
MAPK1	Mitogen-activated protein kinase 1
SASA	Solvent-accessible surface area
PKG	Protein kinase G
CSNK1E	Casein kinase 1 epsilon
